# Chlorido­((*E*)-2-{2-[(2-hy­droxy­benzyl­idene)amino]-2-(pyridin-2-yl)-1-[(pyridin-2-ylmeth­yl)amino]eth­yl}phenolato-κ^4^*N*,*N*′,*N*′′,*O*)copper(II)

**DOI:** 10.1107/S2414314626007169

**Published:** 2026-07-16

**Authors:** Kholood Alkhamis, Yilma Gultneh, Ray J. Butcher

**Affiliations:** a University City, Duba Road, Tabuk, 71491, Saudi Arabia; b Department of Chemistry Howard University, 525 College St NW Washington DC 20059 USA; Vienna University of Technology, Austria

**Keywords:** crystal structure, C—C bond formation, τ parameter, five-coordinate copper complex, Schiff base ligand

## Abstract

The title complex, [Cu(C_26_H_23_N_4_O_2_)Cl], has resulted from C—C bond formation during synthesis. The crystal structure comprises a tetra­dentate ligand, (*E*)-2-{2-[(2-hy­droxy­benzyl­idene)amino]-2-(pyridin-2-yl)-1-[(pyridin-2-ylmeth­yl)amino]­eth­yl}phenolato, binding through three N and one O atom, and an additional chlorido ligand. The coordination environment of the central copper(II) atom is slightly distorted square-pyramidal with a geometry index *τ*_5_ of 0.034.

## Abstract 2

The title copper(II) complex shows the central Cu^II^ cation to be in a slightly distorted square-pyramidal coordination by the [ClN_3_O] coordination set.

## Graphical abstract

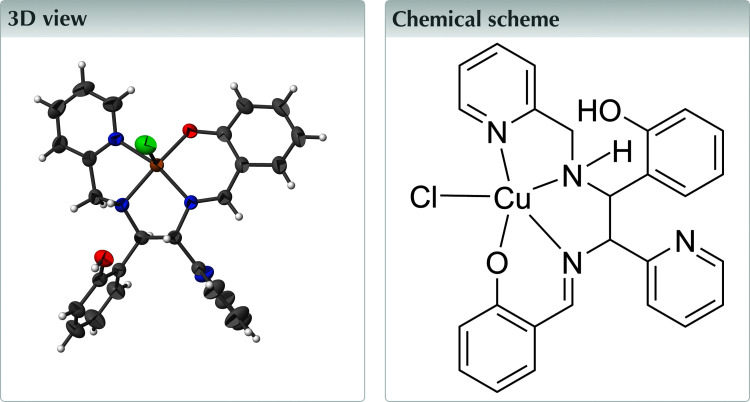

## Structure description

**Figure 1:**
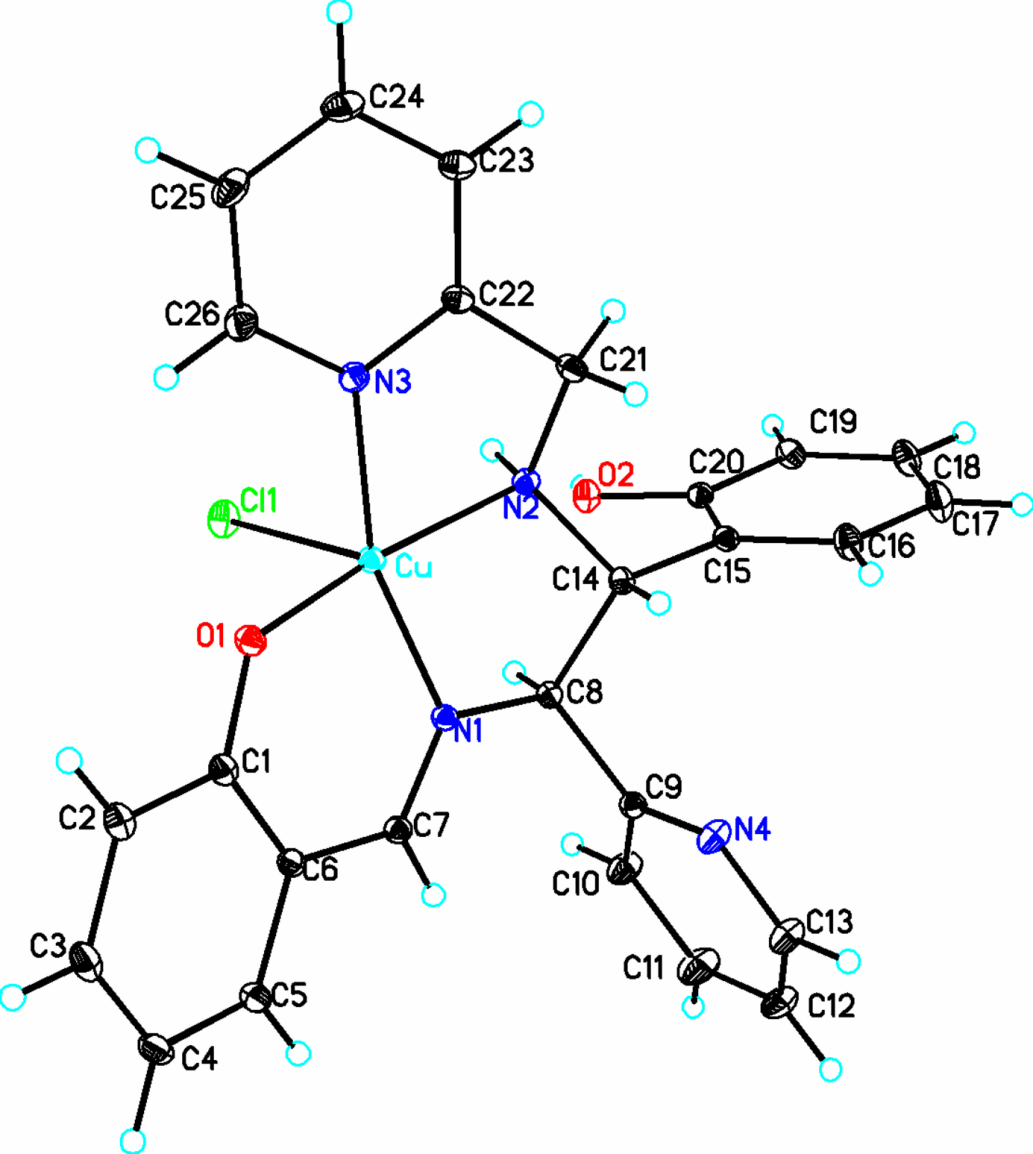
The mol­ecular structure of **1** with the atom labelling; displacement ellipsoids are shown at the 30% probability level.

The crystal structure of a copper(II) complex with the ligand (*E*)-2-{2-[(2-hy­droxy­benzyl­idene)amino]-2-(pyridin-2-yl)-1-[(pyridin-2-ylmeth­yl)amino]­eth­yl}phenolate (*L*H; *L* = C_26_H_23_N_4_O_2_) deprotonated at one of the phenol OH groups is reported. The complex of composition, Cu*L*Cl, **1**, exhibits a square-pyramidal coordination environment around the Cu^II^ atom with the Cl atom in the axial position and with a *τ_5_* parameter of 0.034 (Addison *et al.*, 1984 ▸). The complex resulted from the reaction of a methano­lic solution of CuCl_2_ with a methano­lic solution of 2-{[(pyridin-2-ylmeth­yl)imino]­meth­yl}phenol combined with one equivalent of tri­ethyl­amine; the resulting solution was refluxed for 20 h. The structure of **1** indicates that there has been C—C bond formation between C8 and C14 (Fig. 1 ▸).

As indicated above, **1** has a slightly distorted square-pyramidal coordination environment around the central Cu^II^ atom (Fig. 1 ▸) with the base consisting of donors O1, N1, N2 and N3, and with Cl1 in the axial position. The ON_3_ coordination set is almost planar (root-mean-square deviation of 0.025 Å) with Cu deviating from this plane by 0.252 (1) Å. The basal bond lengths range from 1.900 (2) for Cu—O1 to 2.022 (2) for Cu—N2 while the axial Cu—Cl bond length is 2.5516 (9) (Table 1 ▸). In an examination of the conformation adopted by the ligand it can be seen that, while O1 and N3 are in the coordination plane of the ON_3_ donor atoms, their six-membered rings are not coplanar and make a dihedral angle of 26.2 (2)°.

**Figure 2:**
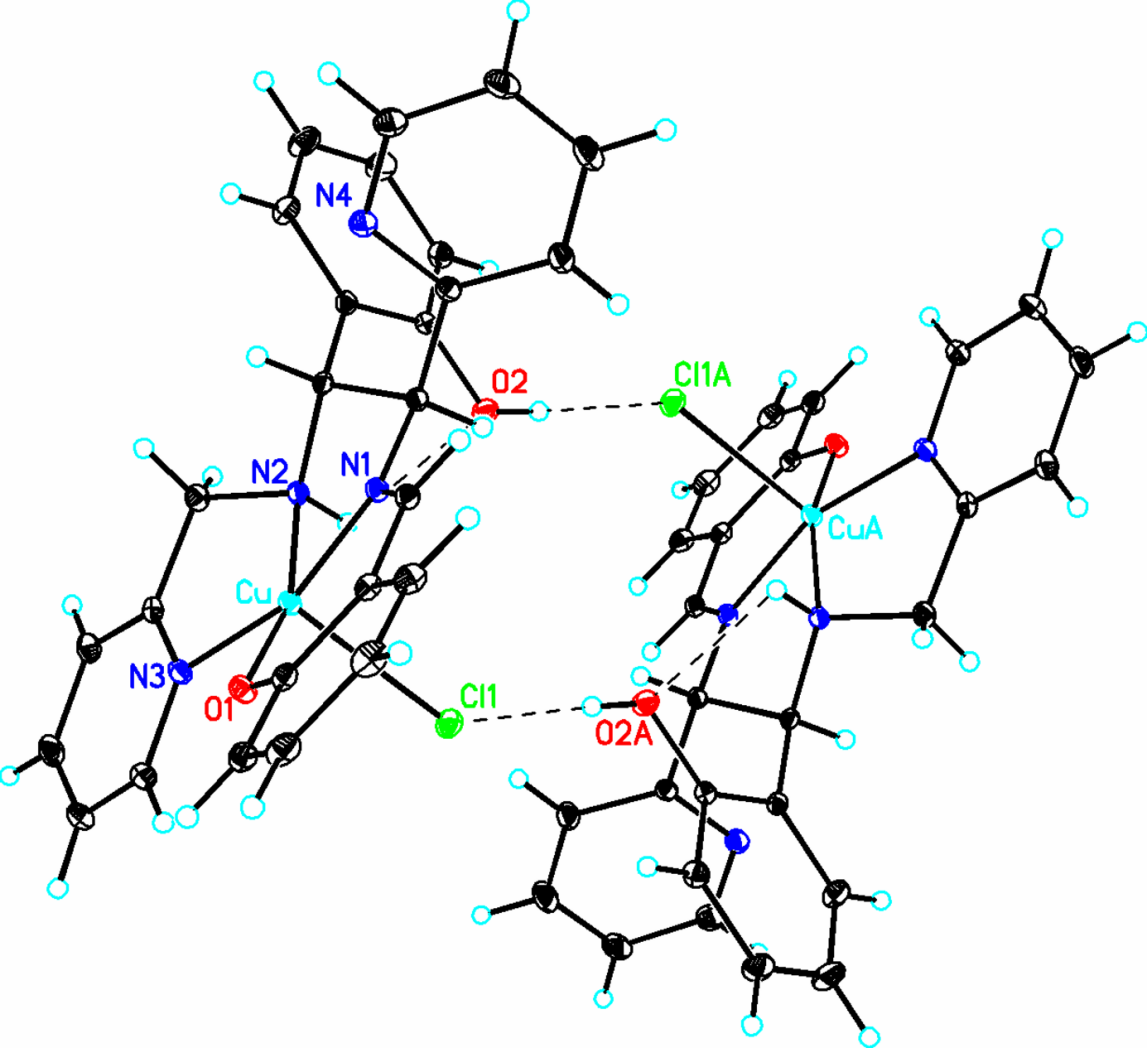
Plot showing the formation of a centrosymmetric dimer by O—H⋯Cl hydrogen bonds (shown as dashed lines). Displacement ellipsoids are shown at the 30% probability level. [Symmetry code: (A) −*x*, −*y* + 1, −*z* + 2].

**Figure 3:**
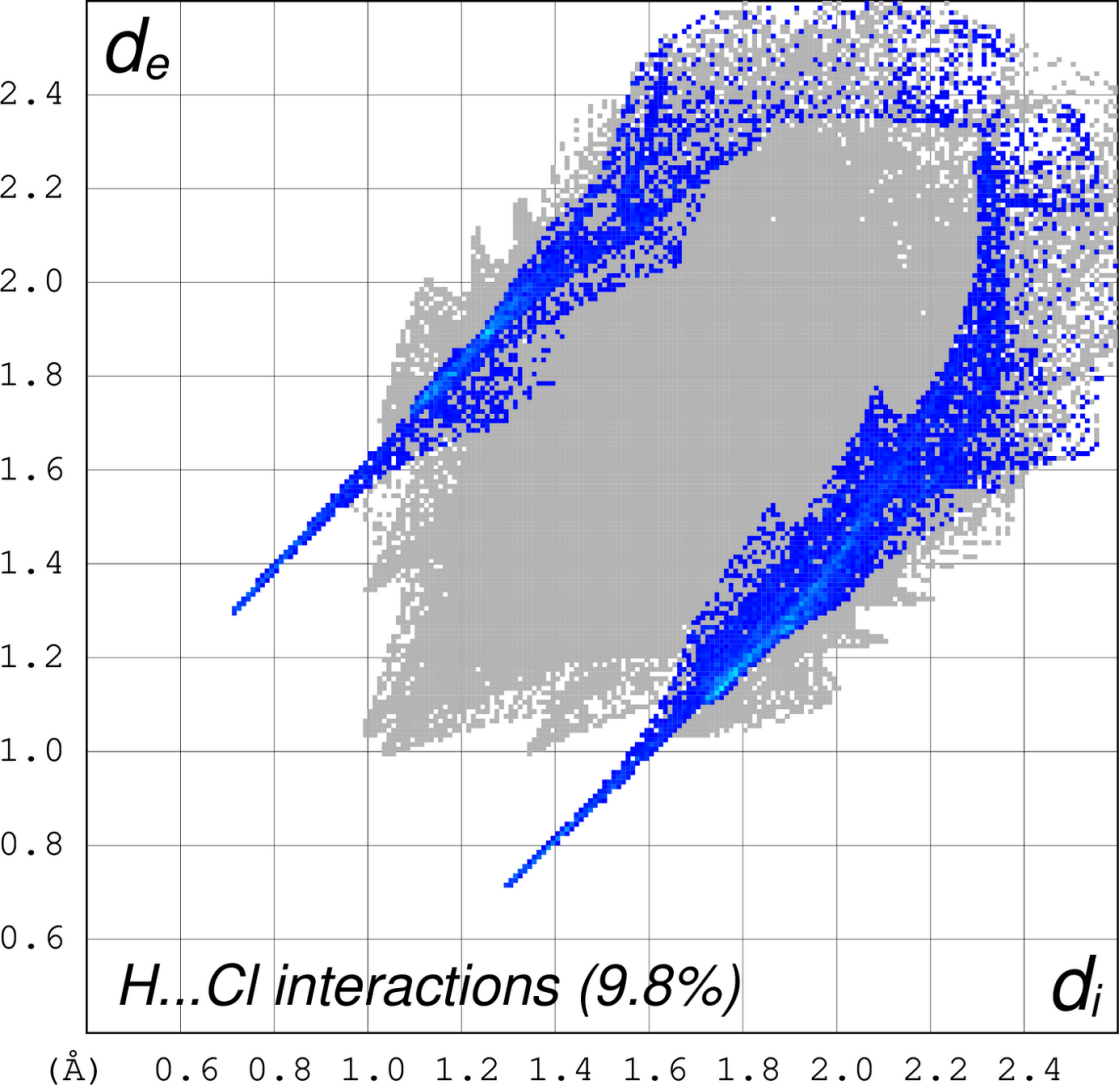
Hirshfeld fingerprint plot showing the O—H⋯Cl inter­actions as prominent spikes.

**Figure 4:**
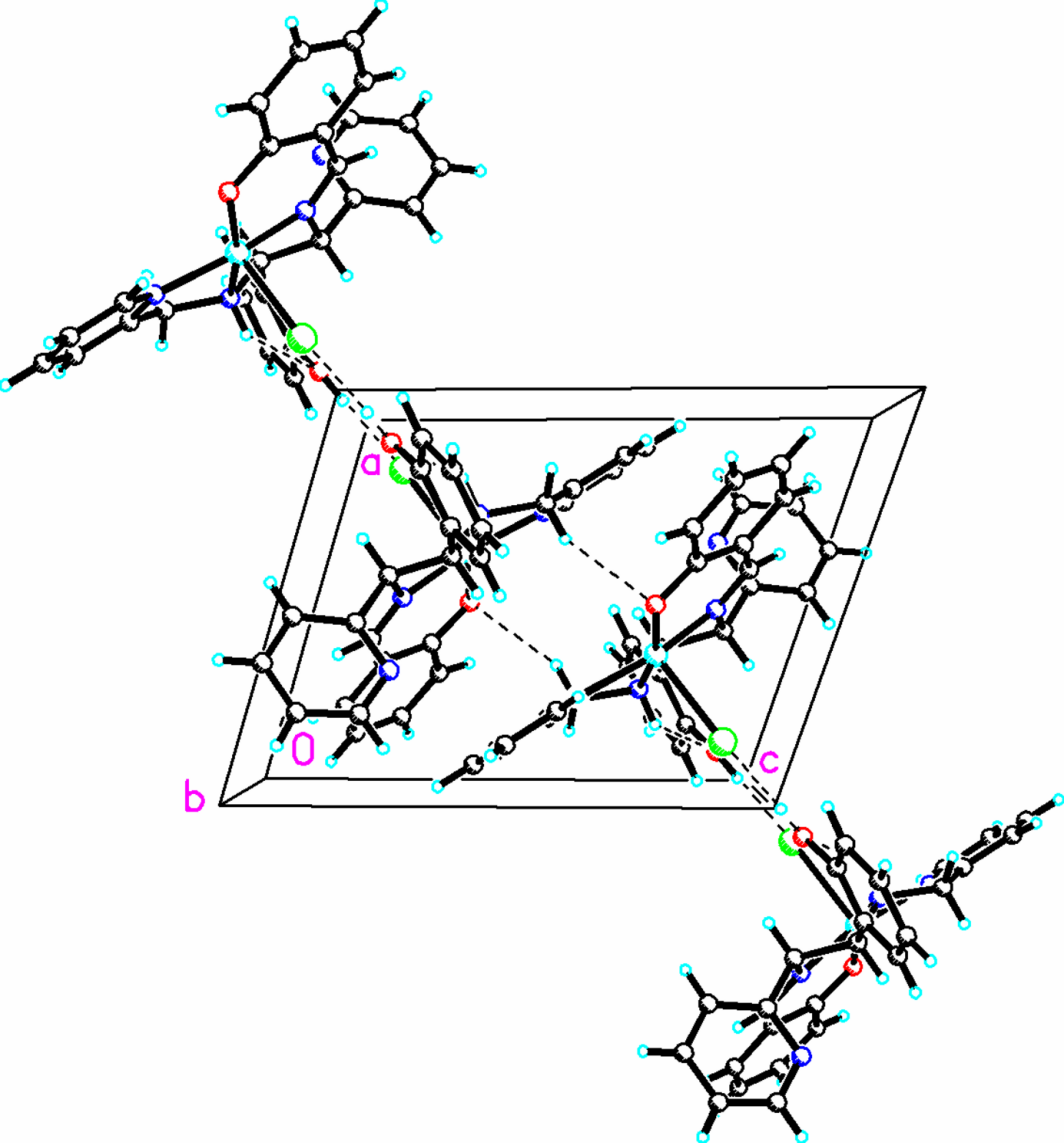
Packing plot of **1** with the mol­ecules linked by various hydrogen-bonding inter­actions (dashed lines).

There is a strong hydrogen-bonding inter­action between the O—H and Cl moieties of adjoining mol­ecules, leading to the formation of a centrosymmetric dimer (Table 2 ▸, Fig. 2 ▸). This is also illustrated in the Hirshfeld fingerprint plot as prominent spikes (Fig. 3 ▸). There is also an intra­molecular N—H⋯O hydrogen bond (Table 2 ▸). The packing of the mol­ecules in the unit cell is shown in Fig. 4 ▸.

A search of the Cambridge Structural Database (CSD version 6.01, update of February 2026; Groom *et al.*, 2016 ▸) for copper(II) compounds with similar coordination sets resulted in five hits (AHEQUA, Plaul *et al.*, 2009 ▸; BUQMAD, Keypour *et al.*, 2015 ▸; HUQHIK, He *et al.*, 2003 ▸; TIDTAF, Nieger, *et al.*, 2023 ▸; WECHAN, Li *et al.*, 2000 ▸) with bond lengths and angles around the central Cu^II^ atom comparable with the title complex.

## Synthesis and crystallization

A solution of 1.66 g (16.37 mmole) of 2-amino­methyl­pyridine in 10 ml of methanol was added to a solution of 2.00 g (16.37 mmole) of salicyl­aldehyde in 10 ml of methanol, and a yellow solution was obtained. The solution was stirred and refluxed overnight for 20 h and then the solvent was removed by rotary evaporation.

The ligand (1.914 g. 8.5 mmole) was dissolved in 15 ml methanol and mixed with tri­ethyl­amine (0.84 ml, 8.5 mmole). To this mixture was added a methanol solution of CuCl_2_ (0.578 g, 4.3 mmole). The dark-blue solution was refluxed and stirred overnight for 20 h. The solvent was reduced by rotary evaporation and the blue precipitate formed was filtered, washed with methanol and diethyl ether and dried in a desiccator. Crystals were grown by diffusion of diethyl ether into an aceto­nitrile solution of the complex.

## Refinement

Crystal data, data collection and structure refinement details are summarized in Table 3 ▸.

## Supplementary Material

Crystal structure: contains datablock(s) I. DOI: 10.1107/S2414314626007169/wm4253sup1.cif

Structure factors: contains datablock(s) I. DOI: 10.1107/S2414314626007169/wm4253Isup2.hkl

CCDC reference: 2572974

Additional supporting information:  crystallographic information; 3D view; checkCIF report

## Figures and Tables

**Table 1:** Selected geometric parameters (Å, °)

Cu—O1	1.900 (2)	Cu—N2	2.022 (2)
Cu—N1	1.943 (2)	Cu—Cl1	2.5516 (9)
Cu—N3	2.009 (2)		
			
O1—Cu—N1	95.67 (9)	N3—Cu—N2	81.68 (10)
O1—Cu—N3	95.01 (9)	O1—Cu—Cl1	99.14 (7)
N1—Cu—N3	161.64 (10)	N1—Cu—Cl1	98.61 (7)
O1—Cu—N2	163.69 (10)	N3—Cu—Cl1	94.38 (8)
N1—Cu—N2	83.90 (9)	N2—Cu—Cl1	97.04 (8)

**Table 2:** Hydrogen-bond geometry (Å, °)

*D*—H⋯*A*	*D*—H	H⋯*A*	*D*⋯*A*	*D*—H⋯*A*
O2—H2*O*⋯Cl1^i^	0.85 (4)	2.16 (4)	3.005 (3)	177 (4)
N2—H2*N*⋯O2	0.89 (3)	2.38 (3)	2.898 (3)	117 (2)
C8—H8*A*⋯O2	0.98	2.46	3.064 (4)	119
C19—H19*A*⋯Cl1^i^	0.93	2.97	3.627 (3)	129
C21—H21*B*⋯O1^ii^	0.97	2.45	3.277 (4)	143

Symmetry codes: (i) 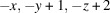; (ii) 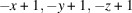.

**Table 3:** Experimental details

Crystal data	
Chemical formula	[CuC_26_H_23_N_4_O_2_)Cl]
*M*_r_	522.47
Crystal system, space group	Triclinic, *P* 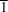
Temperature (K)	296
*a*, *b*, *c* (Å)	9.8728 (16), 11.525 (3), 11.837 (2)
α, β, γ (°)	85.846 (5), 71.992 (3), 66.799 (3)
*V* (Å^3^)	1175.4 (4)
*Z*	2
Radiation type	Mo *K*α
μ (mm^−1^)	1.07
Crystal size (mm)	0.37 × 0.29 × 0.08

Data collection	
Diffractometer	Bruker APEXII CCD
Absorption correction	Multi-scan (*SADABS*; Krause *et al.*, 2015 ▸)
*T*_min_, *T*_max_	0.605, 0.745
No. of measured, independent and observed [*I* > 2σ(*I*)] reflections	11006, 4331, 3578
*R*_int_	0.038
(sin θ/λ)_max_ (Å^−1^)	0.606

Refinement	
*R*[*F*^2^ > 2σ(*F*^2^)], *wR*(*F*^2^), *S*	0.048, 0.132, 1.05
No. of reflections	4331
No. of parameters	315
H-atom treatment	H atoms treated by a mixture of independent and constrained refinement
Δρ_max_, Δρ_min_ (e Å^−3^)	1.34, −0.43

Computer programs: *APEX2* and *SAINT* (Bruker, 2005 ▸), *SHELXT* (Sheldrick, 2015*a* ▸), *SHELXL* (Sheldrick, 2015*b* ▸), *SHELXTL* (Sheldrick, 2008 ▸) and *publCIF* (Westrip, 2010 ▸).

## full crystallographic data

### Chlorido((E)-2-{2-[(2-hydroxybenzylidene)amino]-2-(pyridin-2-yl)-1-[(pyridin-2-ylmethyl)amino]ethyl}phenolato-κ4N,N',N'',O)copper(II) Crystal data

**Table:**

[CuC_26_H_23_N_4_O_2_)Cl]	*Z* = 2
*M**_r_* = 522.47	*F*(000) = 538
Triclinic, *P*1	*D*_x_ = 1.476 Mg m^−^^3^
*a* = 9.8728 (16) Å	Mo *K*α radiation, λ = 0.71073 Å
*b* = 11.525 (3) Å	Cell parameters from 4491 reflections
*c* = 11.837 (2) Å	θ = 2.4–25.4°
α = 85.846 (5)°	µ = 1.07 mm^−^^1^
β = 71.992 (3)°	*T* = 296 K
γ = 66.799 (3)°	Plate, pale blue
*V* = 1175.4 (4) Å^3^	0.37 × 0.29 × 0.08 mm

### Chlorido((E)-2-{2-[(2-hydroxybenzylidene)amino]-2-(pyridin-2-yl)-1-[(pyridin-2-ylmethyl)amino]ethyl}phenolato-κ4N,N',N'',O)copper(II) Data collection

**Table:**

Bruker APEXII CCD diffractometer	3578 reflections with *I* > 2σ(*I*)
ω and φ scans	*R*_int_ = 0.038
Absorption correction: multi-scan (*SADABS*; Krause *et al.*, 2015)	θ_max_ = 25.5°, θ_min_ = 1.8°
*T*_min_ = 0.605, *T*_max_ = 0.745	*h* = −11→11
11006 measured reflections	*k* = −13→13
4331 independent reflections	*l* = −14→14

### Chlorido((E)-2-{2-[(2-hydroxybenzylidene)amino]-2-(pyridin-2-yl)-1-[(pyridin-2-ylmethyl)amino]ethyl}phenolato-κ4N,N',N'',O)copper(II) Refinement

**Table:**

Refinement on *F*^2^	Primary atom site location: dual
Least-squares matrix: full	Secondary atom site location: difference Fourier map
*R*[*F*^2^ > 2σ(*F*^2^)] = 0.048	Hydrogen site location: mixed
*wR*(*F*^2^) = 0.132	H atoms treated by a mixture of independent and constrained refinement
*S* = 1.05	*w* = 1/[σ^2^(*F*_o_^2^) + (0.0895*P*)^2^ + 0.0616*P*] where *P* = (*F*_o_^2^ + 2*F*_c_^2^)/3
4331 reflections	(Δ/σ)_max_ = 0.001
315 parameters	Δρ_max_ = 1.34 e Å^−^^3^
0 restraints	Δρ_min_ = −0.43 e Å^−^^3^

### Chlorido((E)-2-{2-[(2-hydroxybenzylidene)amino]-2-(pyridin-2-yl)-1-[(pyridin-2-ylmethyl)amino]ethyl}phenolato-κ4N,N',N'',O)copper(II) Special details

**Table:**

Geometry. All esds (except the esd in the dihedral angle between two l.s. planes) are estimated using the full covariance matrix. The cell esds are taken into account individually in the estimation of esds in distances, angles and torsion angles; correlations between esds in cell parameters are only used when they are defined by crystal symmetry. An approximate (isotropic) treatment of cell esds is used for estimating esds involving l.s. planes.
Refinement. Hydrogen atoms attached to O and N atoms involved in hydrogen-bonding were refined freely while the remaining hydrogen atoms were included in their calculated positions as a riding model.

### Chlorido((E)-2-{2-[(2-hydroxybenzylidene)amino]-2-(pyridin-2-yl)-1-[(pyridin-2-ylmethyl)amino]ethyl}phenolato-κ4N,N',N'',O)copper(II) Fractional atomic coordinates and isotropic or equivalent isotropic displacement parameters (Å^2^)

**Table:**

	*x*	*y*	*z*	*U*_iso_*/*U*_eq_	
Cu	0.36805 (4)	0.55198 (3)	0.71051 (3)	0.03228 (15)	
Cl1	0.14882 (10)	0.69172 (8)	0.88485 (7)	0.0505 (2)	
O1	0.4969 (2)	0.6446 (2)	0.67674 (19)	0.0386 (5)	
O2	0.1014 (3)	0.3258 (2)	0.9007 (2)	0.0437 (5)	
H2O	0.028 (5)	0.324 (4)	0.961 (4)	0.068 (13)*	
N1	0.4844 (3)	0.4256 (2)	0.8001 (2)	0.0308 (5)	
N2	0.2754 (3)	0.4254 (2)	0.7035 (2)	0.0312 (5)	
H2N	0.182 (4)	0.460 (3)	0.757 (3)	0.032 (8)*	
N3	0.2608 (3)	0.6326 (2)	0.5887 (2)	0.0371 (6)	
N4	0.6651 (3)	0.1333 (3)	0.7720 (2)	0.0481 (7)	
C1	0.6052 (3)	0.6280 (3)	0.7261 (3)	0.0355 (7)	
C2	0.6867 (4)	0.7080 (3)	0.6962 (3)	0.0434 (7)	
H2A	0.661014	0.771253	0.643881	0.052*	
C3	0.8044 (4)	0.6946 (3)	0.7428 (3)	0.0505 (9)	
H3A	0.856228	0.748761	0.721114	0.061*	
C4	0.8459 (4)	0.6024 (3)	0.8208 (3)	0.0511 (9)	
H4A	0.924444	0.594607	0.852068	0.061*	
C5	0.7699 (4)	0.5224 (3)	0.8517 (3)	0.0442 (8)	
H5A	0.797988	0.460208	0.904372	0.053*	
C6	0.6501 (3)	0.5316 (3)	0.8058 (3)	0.0343 (6)	
C7	0.5898 (3)	0.4352 (3)	0.8366 (3)	0.0341 (6)	
H7A	0.629939	0.374835	0.886977	0.041*	
C8	0.4289 (3)	0.3220 (3)	0.8335 (2)	0.0310 (6)	
H8A	0.336631	0.351584	0.903442	0.037*	
C9	0.5471 (3)	0.2021 (3)	0.8623 (3)	0.0338 (6)	
C10	0.5314 (4)	0.1683 (3)	0.9786 (3)	0.0556 (9)	
H10A	0.446990	0.217510	1.040642	0.067*	
C11	0.6450 (5)	0.0591 (4)	1.0006 (4)	0.0754 (13)	
H11A	0.637848	0.034243	1.078080	0.090*	
C12	0.7656 (5)	−0.0107 (4)	0.9088 (4)	0.0626 (10)	
H12A	0.843208	−0.083875	0.921546	0.075*	
C13	0.7708 (4)	0.0290 (4)	0.7969 (4)	0.0577 (9)	
H13A	0.853630	−0.019904	0.733782	0.069*	
C14	0.3807 (3)	0.2995 (3)	0.7274 (2)	0.0301 (6)	
H14A	0.473957	0.271585	0.658173	0.036*	
C15	0.3125 (3)	0.2008 (3)	0.7420 (2)	0.0318 (6)	
C16	0.3869 (4)	0.0914 (3)	0.6693 (3)	0.0489 (8)	
H16A	0.481829	0.077628	0.612432	0.059*	
C17	0.3238 (4)	0.0016 (4)	0.6790 (4)	0.0625 (11)	
H17A	0.376193	−0.071679	0.629425	0.075*	
C18	0.1826 (4)	0.0215 (4)	0.7628 (4)	0.0571 (9)	
H18A	0.139959	−0.038619	0.769465	0.069*	
C19	0.1042 (4)	0.1301 (3)	0.8369 (3)	0.0423 (7)	
H19A	0.008134	0.143975	0.892228	0.051*	
C20	0.1706 (3)	0.2192 (3)	0.8280 (2)	0.0332 (6)	
C21	0.2459 (4)	0.4299 (3)	0.5877 (3)	0.0393 (7)	
H21A	0.161729	0.403345	0.595986	0.047*	
H21B	0.337575	0.372490	0.529480	0.047*	
C22	0.2046 (3)	0.5626 (3)	0.5460 (2)	0.0354 (7)	
C23	0.1198 (4)	0.6093 (3)	0.4679 (3)	0.0461 (8)	
H23A	0.083269	0.558695	0.438360	0.055*	
C24	0.0894 (4)	0.7322 (4)	0.4339 (3)	0.0570 (9)	
H24A	0.033653	0.765234	0.380354	0.068*	
C25	0.1441 (4)	0.8046 (4)	0.4813 (3)	0.0581 (10)	
H25A	0.122561	0.888307	0.461969	0.070*	
C26	0.2292 (4)	0.7527 (3)	0.5563 (3)	0.0498 (8)	
H26A	0.267070	0.801782	0.586498	0.060*	

### Chlorido((E)-2-{2-[(2-hydroxybenzylidene)amino]-2-(pyridin-2-yl)-1-[(pyridin-2-ylmethyl)amino]ethyl}phenolato-κ4N,N',N'',O)copper(II) Atomic displacement parameters (Å^2^)

**Table:**

	*U*^11^	*U*^22^	*U*^33^	*U*^12^	*U*^13^	*U*^23^
Cu	0.0387 (2)	0.0327 (2)	0.0312 (2)	−0.01685 (17)	−0.01561 (16)	0.00614 (15)
Cl1	0.0474 (5)	0.0503 (5)	0.0419 (5)	−0.0184 (4)	0.0031 (4)	−0.0044 (4)
O1	0.0445 (12)	0.0400 (12)	0.0404 (11)	−0.0237 (10)	−0.0174 (10)	0.0100 (9)
O2	0.0409 (12)	0.0413 (13)	0.0435 (13)	−0.0186 (10)	−0.0011 (10)	−0.0033 (10)
N1	0.0323 (12)	0.0324 (13)	0.0295 (12)	−0.0134 (10)	−0.0115 (10)	0.0031 (10)
N2	0.0319 (13)	0.0331 (13)	0.0272 (12)	−0.0118 (11)	−0.0089 (10)	0.0034 (10)
N3	0.0442 (15)	0.0352 (14)	0.0345 (13)	−0.0173 (11)	−0.0145 (11)	0.0079 (11)
N4	0.0446 (16)	0.0409 (16)	0.0431 (16)	−0.0052 (13)	−0.0082 (13)	0.0046 (12)
C1	0.0329 (15)	0.0379 (16)	0.0326 (15)	−0.0145 (13)	−0.0037 (12)	−0.0051 (12)
C2	0.0469 (18)	0.0448 (18)	0.0409 (17)	−0.0245 (15)	−0.0068 (14)	−0.0030 (14)
C3	0.0452 (19)	0.052 (2)	0.057 (2)	−0.0278 (17)	−0.0044 (16)	−0.0133 (17)
C4	0.0388 (18)	0.061 (2)	0.060 (2)	−0.0215 (17)	−0.0174 (16)	−0.0110 (18)
C5	0.0402 (17)	0.0447 (19)	0.0506 (19)	−0.0149 (15)	−0.0182 (15)	−0.0051 (15)
C6	0.0304 (15)	0.0365 (16)	0.0343 (15)	−0.0115 (12)	−0.0086 (12)	−0.0039 (12)
C7	0.0353 (15)	0.0346 (16)	0.0316 (14)	−0.0117 (13)	−0.0121 (12)	0.0021 (12)
C8	0.0346 (15)	0.0320 (15)	0.0283 (14)	−0.0150 (12)	−0.0098 (12)	0.0034 (11)
C9	0.0341 (15)	0.0339 (16)	0.0363 (15)	−0.0157 (13)	−0.0120 (13)	0.0046 (12)
C10	0.058 (2)	0.054 (2)	0.0387 (18)	−0.0063 (18)	−0.0152 (17)	0.0085 (16)
C11	0.085 (3)	0.071 (3)	0.052 (2)	−0.009 (2)	−0.031 (2)	0.024 (2)
C12	0.060 (2)	0.048 (2)	0.072 (3)	−0.0067 (18)	−0.031 (2)	0.0167 (19)
C13	0.047 (2)	0.047 (2)	0.061 (2)	−0.0027 (16)	−0.0128 (17)	0.0026 (17)
C14	0.0316 (14)	0.0317 (15)	0.0246 (13)	−0.0124 (12)	−0.0049 (11)	−0.0009 (11)
C15	0.0348 (15)	0.0337 (15)	0.0287 (14)	−0.0140 (12)	−0.0112 (12)	0.0017 (12)
C16	0.0477 (19)	0.050 (2)	0.0457 (19)	−0.0239 (16)	−0.0012 (15)	−0.0110 (16)
C17	0.067 (2)	0.055 (2)	0.067 (2)	−0.031 (2)	−0.007 (2)	−0.0203 (19)
C18	0.068 (2)	0.053 (2)	0.065 (2)	−0.039 (2)	−0.019 (2)	−0.0038 (18)
C19	0.0424 (17)	0.0469 (19)	0.0435 (18)	−0.0265 (15)	−0.0098 (14)	0.0051 (14)
C20	0.0366 (16)	0.0328 (16)	0.0296 (14)	−0.0125 (13)	−0.0113 (12)	0.0038 (12)
C21	0.0472 (18)	0.0456 (18)	0.0357 (16)	−0.0237 (15)	−0.0204 (14)	0.0061 (13)
C22	0.0352 (15)	0.0443 (18)	0.0266 (14)	−0.0161 (13)	−0.0091 (12)	0.0037 (12)
C23	0.0480 (19)	0.057 (2)	0.0371 (17)	−0.0205 (16)	−0.0195 (15)	0.0089 (15)
C24	0.061 (2)	0.065 (2)	0.0415 (19)	−0.0164 (19)	−0.0260 (18)	0.0176 (17)
C25	0.073 (3)	0.042 (2)	0.049 (2)	−0.0139 (18)	−0.0199 (19)	0.0167 (16)
C26	0.068 (2)	0.0424 (19)	0.0410 (18)	−0.0229 (17)	−0.0187 (17)	0.0081 (15)

### Chlorido((E)-2-{2-[(2-hydroxybenzylidene)amino]-2-(pyridin-2-yl)-1-[(pyridin-2-ylmethyl)amino]ethyl}phenolato-κ4N,N',N'',O)copper(II) Geometric parameters (Å, º)

**Table:**

Cu—O1	1.900 (2)	C9—C10	1.382 (4)
Cu—N1	1.943 (2)	C10—C11	1.390 (5)
Cu—N3	2.009 (2)	C10—H10A	0.9300
Cu—N2	2.022 (2)	C11—C12	1.348 (6)
Cu—Cl1	2.5516 (9)	C11—H11A	0.9300
O1—C1	1.315 (3)	C12—C13	1.363 (5)
O2—C20	1.359 (4)	C12—H12A	0.9300
O2—H2O	0.85 (4)	C13—H13A	0.9300
N1—C7	1.288 (4)	C14—C15	1.510 (4)
N1—C8	1.482 (3)	C14—H14A	0.9800
N2—C21	1.480 (4)	C15—C16	1.381 (4)
N2—C14	1.483 (4)	C15—C20	1.400 (4)
N2—H2N	0.89 (3)	C16—C17	1.386 (5)
N3—C22	1.341 (4)	C16—H16A	0.9300
N3—C26	1.349 (4)	C17—C18	1.380 (5)
N4—C9	1.324 (4)	C17—H17A	0.9300
N4—C13	1.331 (4)	C18—C19	1.383 (5)
C1—C2	1.409 (4)	C18—H18A	0.9300
C1—C6	1.429 (4)	C19—C20	1.402 (4)
C2—C3	1.386 (5)	C19—H19A	0.9300
C2—H2A	0.9300	C21—C22	1.508 (4)
C3—C4	1.378 (5)	C21—H21A	0.9700
C3—H3A	0.9300	C21—H21B	0.9700
C4—C5	1.368 (5)	C22—C23	1.377 (4)
C4—H4A	0.9300	C23—C24	1.384 (5)
C5—C6	1.412 (4)	C23—H23A	0.9300
C5—H5A	0.9300	C24—C25	1.384 (6)
C6—C7	1.431 (4)	C24—H24A	0.9300
C7—H7A	0.9300	C25—C26	1.357 (5)
C8—C9	1.515 (4)	C25—H25A	0.9300
C8—C14	1.545 (4)	C26—H26A	0.9300
C8—H8A	0.9800		
			
O1—Cu—N1	95.67 (9)	C9—C10—H10A	120.8
O1—Cu—N3	95.01 (9)	C11—C10—H10A	120.8
N1—Cu—N3	161.64 (10)	C12—C11—C10	119.5 (4)
O1—Cu—N2	163.69 (10)	C12—C11—H11A	120.3
N1—Cu—N2	83.90 (9)	C10—C11—H11A	120.3
N3—Cu—N2	81.68 (10)	C11—C12—C13	118.2 (3)
O1—Cu—Cl1	99.14 (7)	C11—C12—H12A	120.9
N1—Cu—Cl1	98.61 (7)	C13—C12—H12A	120.9
N3—Cu—Cl1	94.38 (8)	N4—C13—C12	124.3 (4)
N2—Cu—Cl1	97.04 (8)	N4—C13—H13A	117.9
C1—O1—Cu	124.75 (19)	C12—C13—H13A	117.9
C20—O2—H2O	113 (3)	N2—C14—C15	112.9 (2)
C7—N1—C8	122.5 (2)	N2—C14—C8	105.9 (2)
C7—N1—Cu	124.1 (2)	C15—C14—C8	115.8 (2)
C8—N1—Cu	113.12 (16)	N2—C14—H14A	107.3
C21—N2—C14	114.2 (2)	C15—C14—H14A	107.3
C21—N2—Cu	109.34 (17)	C8—C14—H14A	107.3
C14—N2—Cu	108.23 (16)	C16—C15—C20	118.3 (3)
C21—N2—H2N	105.2 (19)	C16—C15—C14	120.8 (3)
C14—N2—H2N	117.2 (19)	C20—C15—C14	120.8 (2)
Cu—N2—H2N	101.9 (19)	C15—C16—C17	121.7 (3)
C22—N3—C26	118.7 (3)	C15—C16—H16A	119.2
C22—N3—Cu	115.58 (19)	C17—C16—H16A	119.2
C26—N3—Cu	125.4 (2)	C18—C17—C16	119.6 (3)
C9—N4—C13	117.5 (3)	C18—C17—H17A	120.2
O1—C1—C2	118.3 (3)	C16—C17—H17A	120.2
O1—C1—C6	124.7 (3)	C17—C18—C19	120.5 (3)
C2—C1—C6	117.0 (3)	C17—C18—H18A	119.7
C3—C2—C1	121.5 (3)	C19—C18—H18A	119.7
C3—C2—H2A	119.2	C18—C19—C20	119.4 (3)
C1—C2—H2A	119.2	C18—C19—H19A	120.3
C4—C3—C2	121.1 (3)	C20—C19—H19A	120.3
C4—C3—H3A	119.4	O2—C20—C15	117.3 (3)
C2—C3—H3A	119.4	O2—C20—C19	122.2 (3)
C5—C4—C3	119.1 (3)	C15—C20—C19	120.5 (3)
C5—C4—H4A	120.4	N2—C21—C22	109.8 (2)
C3—C4—H4A	120.4	N2—C21—H21A	109.7
C4—C5—C6	121.9 (3)	C22—C21—H21A	109.7
C4—C5—H5A	119.1	N2—C21—H21B	109.7
C6—C5—H5A	119.1	C22—C21—H21B	109.7
C5—C6—C1	119.3 (3)	H21A—C21—H21B	108.2
C5—C6—C7	116.2 (3)	N3—C22—C23	121.7 (3)
C1—C6—C7	124.3 (3)	N3—C22—C21	114.6 (2)
N1—C7—C6	125.1 (3)	C23—C22—C21	123.7 (3)
N1—C7—H7A	117.4	C22—C23—C24	119.3 (3)
C6—C7—H7A	117.4	C22—C23—H23A	120.3
N1—C8—C9	113.9 (2)	C24—C23—H23A	120.3
N1—C8—C14	105.4 (2)	C25—C24—C23	118.4 (3)
C9—C8—C14	111.8 (2)	C25—C24—H24A	120.8
N1—C8—H8A	108.5	C23—C24—H24A	120.8
C9—C8—H8A	108.5	C26—C25—C24	119.6 (3)
C14—C8—H8A	108.5	C26—C25—H25A	120.2
N4—C9—C10	122.3 (3)	C24—C25—H25A	120.2
N4—C9—C8	117.2 (3)	N3—C26—C25	122.3 (3)
C10—C9—C8	120.6 (3)	N3—C26—H26A	118.8
C9—C10—C11	118.4 (3)	C25—C26—H26A	118.8
			
Cu—O1—C1—C2	−177.1 (2)	Cu—N2—C14—C8	44.0 (2)
Cu—O1—C1—C6	4.9 (4)	N1—C8—C14—N2	−51.4 (3)
O1—C1—C2—C3	−178.7 (3)	C9—C8—C14—N2	−175.7 (2)
C6—C1—C2—C3	−0.6 (4)	N1—C8—C14—C15	−177.4 (2)
C1—C2—C3—C4	−0.2 (5)	C9—C8—C14—C15	58.3 (3)
C2—C3—C4—C5	0.5 (5)	N2—C14—C15—C16	122.0 (3)
C3—C4—C5—C6	0.0 (5)	C8—C14—C15—C16	−115.7 (3)
C4—C5—C6—C1	−0.8 (5)	N2—C14—C15—C20	−56.7 (3)
C4—C5—C6—C7	174.5 (3)	C8—C14—C15—C20	65.6 (3)
O1—C1—C6—C5	179.1 (3)	C20—C15—C16—C17	0.7 (5)
C2—C1—C6—C5	1.1 (4)	C14—C15—C16—C17	−178.0 (3)
O1—C1—C6—C7	4.1 (5)	C15—C16—C17—C18	0.3 (6)
C2—C1—C6—C7	−173.9 (3)	C16—C17—C18—C19	−0.1 (6)
C8—N1—C7—C6	178.8 (3)	C17—C18—C19—C20	−1.2 (6)
Cu—N1—C7—C6	−7.6 (4)	C16—C15—C20—O2	178.2 (3)
C5—C6—C7—N1	−177.6 (3)	C14—C15—C20—O2	−3.1 (4)
C1—C6—C7—N1	−2.5 (5)	C16—C15—C20—C19	−2.0 (4)
C7—N1—C8—C9	−26.7 (4)	C14—C15—C20—C19	176.7 (3)
Cu—N1—C8—C9	159.09 (19)	C18—C19—C20—O2	−178.0 (3)
C7—N1—C8—C14	−149.6 (3)	C18—C19—C20—C15	2.3 (5)
Cu—N1—C8—C14	36.2 (2)	C14—N2—C21—C22	−153.9 (2)
C13—N4—C9—C10	−0.4 (5)	Cu—N2—C21—C22	−32.5 (3)
C13—N4—C9—C8	178.8 (3)	C26—N3—C22—C23	1.8 (4)
N1—C8—C9—N4	−73.2 (3)	Cu—N3—C22—C23	175.7 (2)
C14—C8—C9—N4	46.2 (3)	C26—N3—C22—C21	−179.8 (3)
N1—C8—C9—C10	106.1 (3)	Cu—N3—C22—C21	−5.9 (3)
C14—C8—C9—C10	−134.6 (3)	N2—C21—C22—N3	25.6 (4)
N4—C9—C10—C11	0.8 (5)	N2—C21—C22—C23	−156.0 (3)
C8—C9—C10—C11	−178.4 (3)	N3—C22—C23—C24	−1.0 (5)
C9—C10—C11—C12	−0.4 (6)	C21—C22—C23—C24	−179.2 (3)
C10—C11—C12—C13	−0.3 (7)	C22—C23—C24—C25	−0.9 (5)
C9—N4—C13—C12	−0.3 (6)	C23—C24—C25—C26	2.0 (6)
C11—C12—C13—N4	0.7 (7)	C22—N3—C26—C25	−0.7 (5)
C21—N2—C14—C15	−66.3 (3)	Cu—N3—C26—C25	−173.9 (3)
Cu—N2—C14—C15	171.70 (17)	C24—C25—C26—N3	−1.3 (6)
C21—N2—C14—C8	166.0 (2)		

### Chlorido((E)-2-{2-[(2-hydroxybenzylidene)amino]-2-(pyridin-2-yl)-1-[(pyridin-2-ylmethyl)amino]ethyl}phenolato-κ4N,N',N'',O)copper(II) Hydrogen-bond geometry (Å, º)

**Table:**

*D*—H···*A*	*D*—H	H···*A*	*D*···*A*	*D*—H···*A*
O2—H2*O*···Cl1^i^	0.85 (4)	2.16 (4)	3.005 (3)	177 (4)
N2—H2*N*···O2	0.89 (3)	2.38 (3)	2.898 (3)	117 (2)
C8—H8*A*···O2	0.98	2.46	3.064 (4)	119
C19—H19*A*···Cl1^i^	0.93	2.97	3.627 (3)	129
C21—H21*B*···O1^ii^	0.97	2.45	3.277 (4)	143

Symmetry codes: (i) −*x*, −*y*+1, −*z*+2; (ii) −*x*+1, −*y*+1, −*z*+1.
